# Mapping and transcriptomic approches implemented for understanding disease resistance to *Phytophthora cinammomi* in *Castanea sp*

**DOI:** 10.1186/1753-6561-5-S7-O18

**Published:** 2011-09-13

**Authors:** Rita Costa, Carmen Santos, Fábio Tavares, Helena Machado, José Gomes-Laranjo, Thomas Kubisiak, Charles Dana Nelson

**Affiliations:** 1 Molecular Biology Laboratory –L-INIA - INRB I.P. Av da República, 2780-159 Oeiras, Portugal; 2 Centre for Investigation and Agro Environmental and Biological Technologies (CITAB) – Department of Agronomy, University of Trás-os-Montes e Alto Douro, Apt. 1013, 5001-801 Vila Real, Portugal; 3 USDA Forest Service, Southern Research Station, Southern Institute of Forest Genetics, 23332 Old Mississippi Highway 67, Saucier, MS, USA

## 

The European chestnut, *Castanea sativa* Mill, covers a total area of 2.53 million hectares two million of which are chestnut forests, i.e. forests where chestnut is the dominant tree species, being the remaining 0.53 million hectares devoted to fruit production (20.9% of the total chestnut-growing area). Chestnut fruit production in Europe declined considerably during the XX century to the current 200,000 t (almost 300 million euros). This decline arose mainly due to serious diseases and changes in the structure of society. Towards the end of the century, there was a sharp increase in chestnut demand which triggered new planting and the restoration of old orchards throughout Europe, although ink disease (*Phytophthora* spp.), canker blight (*Cryphonectria parasitica* (Murrill) M.E. Barr.) and more recently *Dryocosmus kuriphilus* Yasumatsu, still represent major threats to the species. In Europe, chestnut breeding has been focused on the improvement of cultivars and rootstocks through selection and hybridisation with Asian species resistant to ink disease. Considerable success was obtained using this approach, however little information was acquired until now on the genetic basis of resistance in chestnut.

A set of interspecific crosses were established between European chestnut and Asian species and two separate full-sib pedigrees have been produced, SC (*C. sativa* x *C. crenata*) and SM (*C.sativa* x *C. mollissima*). The goal is to perform DNA marker:trait association analysis to identify QTLs related to ink disease metrics and also to identify putative resistance genes to *P cinnamomi* using a transcriptomic approach. An overview of the study will be presented including the genotyping of the mapping population (parents and progenies) with nuclear SSR markers designed for *Castanea mollissima* and provided by the American team of Fagaceae Genomics Project, for the construction of the first genetic linkage map. The methodologies implemented for the determination of the metrics of resistance of mapping population, for further identification of QTLs, will also be presented.

